# Application of Machine Learning in Food Safety Risk Assessment

**DOI:** 10.3390/foods14234005

**Published:** 2025-11-22

**Authors:** Qingchuan Zhang, Zhe Lu, Zhenqiao Liu, Jialu Li, Mingchao Chang, Min Zuo

**Affiliations:** 1 National Engineering Research Center for Agri-Product Quality Traceability, Beijing Technology and Business University, No. 11 and No. 33 Fucheng Road, Haidian District, Beijing 100048, China; zhangqingchuan@btbu.edu.cn (Q.Z.); 2331101001@st.btbu.edu.cn (Z.L.); 2431061235@st.btbu.edu.cn (Z.L.); 2330702019@st.btbu.edu.cn (J.L.); 2431061064@st.btbu.edu.cn (M.C.); 2 Business School, Beijing Wuzi University, 321 Fuhe Street, Tongzhou District, Beijing 101149, China

**Keywords:** food safety, machine learning, deep learning, globalization

## Abstract

With the increasing globalization of supply chains, ensuring food safety has become more complex, necessitating advanced approaches for risk assessment. This study aims to review the transformative role of machine learning (ML) and deep learning (DL) in enabling intelligent food safety management by efficiently analyzing high-quality and nonlinear data. We systematically summarize recent advances in the application of ML and DL, focusing on key areas such as biotoxin detection, heavy metal contamination, analysis of pesticide and veterinary drug residues, and microbial risk prediction. While traditional algorithms including support vector machines and random forests demonstrate strong performance in classification and risk evaluation, unsupervised methods such as K-means and hierarchical cluster analysis facilitate pattern recognition in unlabeled datasets. Furthermore, novel DL architectures, such as convolutional neural networks, recurrent neural networks, and transformers, enable automated feature extraction and multimodal data integration, substantially improving detection accuracy and efficiency. In conclusion, we recommend future work to emphasize model interpretability, multi-modal data fusion, and integration into HACCP systems, thereby supporting intelligent, interpretable, and real-time food safety management.

## 1. Introduction

Food safety serves as a critical pillar of global public health [1]. Animal-derived foods (ADFPs) and cereal grains, which are fundamental sources of high-quality protein and essential nutrients, are highly susceptible to biological contaminants (e.g., mycotoxins) and chemical hazards (e.g., heavy metals and pesticide residues). These contaminants pose significant risks to human health and can undermine public trust in the food system. Hazards are cross-boundary, primarily including carcinogenic mycotoxins toxins [2], chronic heavy metal contaminants [3], microbial pathogens [4], and endocrine-disrupting residues [5]. These hazards often interact to form complex networks that challenge traditional assessment methods [6]. Existing approaches, relying on statistical models, increasingly struggle to process high-dimensional heterogeneous data and enable real-time early warning [7].

To address these limitations, Machine Learning (ML) offers innovative analytical tools [8,9,10,11]. Traditional algorithms like SVM and Random Forest have successfully applied multi-source data to pollutant detection and risk classification [12,13], while unsupervised methods (e.g., K-Means) provide value in pattern recognition for unlabeled data [14,15]. However, classical ML is limited by its reliance on hand-crafted feature extraction [6,14]. The emergence of DL marks a new phase [16,17]. Convolutional Neural Networks (CNNs) excel in automatic feature extraction from spectral data [18], while Recurrent Neural Networks (RNNs) capture dynamic dependencies in time-series data [19]. Recently, Transformers and Graph Neural Networks (GNN) have pushed boundaries by enabling global context modeling, advancing the field from single-hazard detection to systematic early warning [20,21,22].

The purpose of this paper is to systematically summarize the research status of ML and DL in recent years in the field of food safety risk assessment. To ensure a comprehensive and reproducible review, we conducted a systematic literature review guided by the following research questions: (1) What are the predominant ML and DL algorithms applied in food safety risk assessment? (2) How do these algorithms perform across the four major hazard categories: biotoxins, heavy metal pollution, pesticide and veterinary drug residues, and microbial risks? (3) What are the current challenges and future directions for the field?

First, we move beyond a general discussion by providing a systematic, side-by-side comparison of algorithmic performance across the four most critical food hazard categories—mycotoxins, heavy metals, pesticide and veterinary drug residues, and microbial risks—offering a clear roadmap for selecting the optimal tool for specific safety challenges. Second, we place a strong emphasis on the latest advancements in deep learning architectures, particularly Transformer-based models and their hybrid variants (e.g., CNN-Transformer), which are reshaping the field but have not been comprehensively covered in earlier summaries. Finally, and most importantly, our review extends beyond a technological survey to provide a critical discussion on translational challenges, such as model interpretability and data scarcity, and proposes concrete pathways for integrating these intelligent systems into existing food safety management frameworks like HACCP. This combination of a structured, multi-hazard analysis, a focus on cutting-edge models, and a critical perspective on implementation barriers provides a timely and actionable resource for advancing the field.

Our analysis is based on a systematic search of the Scopus, Web of Science, and PubMed databases, following the established practice of previous reviews in this domain. The search strategy combined keywords for machine learning (“unsupervised learning” OR “supervised learning” OR “deep learning” OR “machine learning”), food products (“dairy product” OR “meat” OR “vegetable” OR “fish” OR “fruit”), and specific food risks (“biotoxin”, “heavy metal pollution”, “pesticide and veterinary drug residue”, and “microbial risk”). This process initially identified 1000 records. After duplicate removal and application of inclusion/exclusion criteria (e.g., peer-reviewed articles published between 2015 and 2025, focusing on primary applications of ML/DL in the targeted risk categories), a total of 132 studies were selected for in-depth analysis and data extraction. The extracted information, including the food product, data type, algorithm, and key findings, forms the basis of the comparative tables and discussion in this review.

## 2. Unsupervised Machine Learning Algorithm

**Figure 1:**
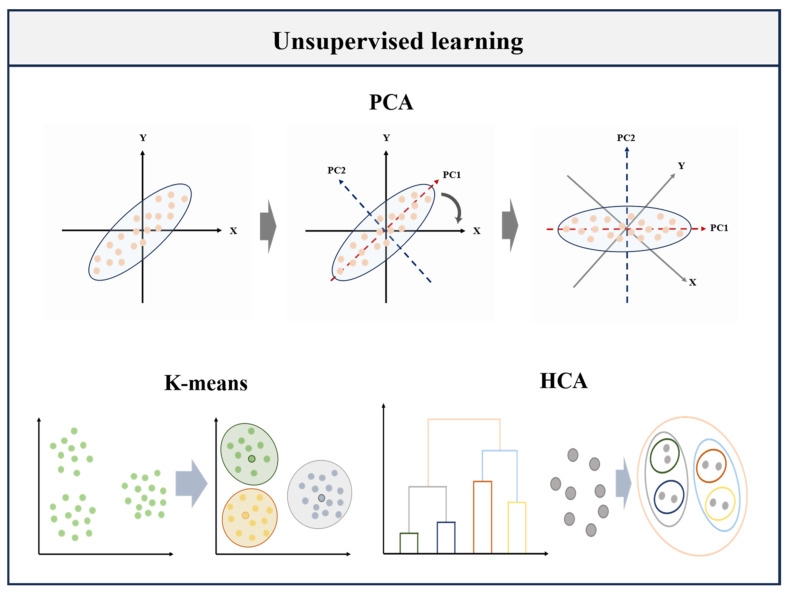
Schematic diagram of unsupervised learning (PCA, K-means, HCA) algorithm.

Unsupervised Learning refers to a class of ML algorithms whose purpose is to detect structures, patterns, and relationships from unlabeled datasets (Figure 1) [23]. Without relying on pre-defined output labels, these methods excel at exploratory data analysis, with their primary tasks being clustering (e.g., grouping similar data points) and dimensionality reduction. In addition to discovering the data structure and finding rules, unsupervised learning methods can also be used in dimensionality reduction and are usually regarded as an auxiliary measure to improve efficiency in data visualization or build later analysis/prediction models.

### 2.1. Hierarchical Cluster Analysis (HCA) Algorithm

Hierarchical cluster analysis (HCA) is a distance-based, unsupervised approach that commonly analyzes multivariate data [24]. This method uses a distance matrix to quantify the pair difference between observations. HCA iteratively combines the two most similar objects, which initially are individual observations (pairs), and in subsequent steps are clusters of observations [25]. This process takes until all data points are grouped, creating a hierarchy that represents the organization of the record structure. The results are typically visualized with a tree chart that shows the hierarchy and arrangement of the result cluster [26]. In the field of food safety, clustering algorithms have been used to identify foodborne pathogens [27]. For example, HCA was successfully applied as a tool for differentiating among several groups of foodborne pathogens [28].

In addition, HCA can be combined with other algorithms for food risk assessment. Previous studies have combined HCA with LDA to identify and detect heavy metal particles and pesticide residues in substrates such as soil, tap water, lettuce and apples [29]. Initially, HCA clustered similar samples effectively without supervision, revealed potential patterns in unmarked data, and provided information on differences between sample groups for downstream classification models [30]. LDA took advantage of these differences and used their advantages in monitoring classification tasks to achieve greater accuracy in analyte identification and quantitative evaluation [31]. This combined method can significantly improve the accuracy and reliability of identifying and distinguishing complex samples and thus the risk assessment for improving food safety [32].

### 2.2. K-Means Algorithm

K-means is a distance-based, unsupervised cluster algorithm, which mainly seeks to automatically divide similar samples into different classes/clusters [33]. Firstly, it randomly sets several initial cluster centroids; then, it iteratively computes the distance between all samples and the centroid coordinates and classifies them according to the closest distance. Next, calculate the centroid coordinates again based on the newly classified dataset until there is no change in the classification results or a fixed number of iterations are performed. Because of its simple principle, fast speed, and high-efficiency feature extraction ability, the K-means clustering algorithm plays a key role in food safety risk prediction and evaluation work. In a related research conducted earlier by other researchers, whose purpose was to predict and identify the infection intensity of Aflatoxin in Pistachio, K-means clustering algorithms were also used for data dimensional compression processing of hyperspectral imagers [34]. As an unsupervised classifier, the model successfully discovered the cluster distribution rules hidden beneath such large-scale image information, so as to effectively separate positive cases from negative samples.

In another study, K-means clustering is used as an important data preprocessing technology in image segmentation to detect symptoms of fungal disease on the surface of potato leaves [35]. The algorithm partitioned the leaf images into distinct clusters based on pixel color and intensity features, effectively separating regions of interest, which consisted of diseased or healthy leaf areas, from the background [36]. This process serves to accentuate disease-related features while concurrently mitigating background noise and irrelevant information. Together with later data enhancement processing, this treatment greatly promotes feature extraction in subsequent networks, which helps improve prediction accuracy.

### 2.3. Principal Component Analysis (PCA) Algorithm

Principal component analysis (PCA) is an unsupervised analytical technique widely applied for dimensionality reduction in multivariate datasets [37]. Its primary purpose is to simplify complex high-dimensional data while retaining as much of the original information as possible. PCA operates by transforming a set of correlated variables into a smaller number of uncorrelated principal components, following the principle of maximizing the variance captured by each successive component. Through this process, PCA eliminates redundant information and reveals the underlying structure of the data. It helps to interpret the covariance relationships among variables and identify the key factors that contribute most to the overall variability within the dataset.

For food safety risk prediction, PCA is usually used as a pre-processing link before modeling to conduct dimensional reduction processing on the original high-dimensional data and extract core features. Combined with data-driven models that can well characterize the nonlinear relationship between input and output variables, PCA can make the data analysis deeper and more accurate [38,39]. For example, in a study aiming to identify fungal contamination in rice samples, PCA was first applied to the hyperspectral data of rice to explore the intrinsic relationships among wavelength variables [40]. This reduces dimensions by retaining most of the variation information while removing data redundancy, so as to provide a more compact and effective feature representation for the later training of the Gaussian SVM model for sample prediction.

## 3. Machine Learning Algorithms

ML encompasses a broad family of computational methods designed to learn patterns, representations, and predictive rules from data. Depending on the availability of annotated information and the nature of the task, ML methods can be broadly categorized into unsupervised learning, supervised learning, and deep learning. Unsupervised learning focuses on discovering intrinsic structures, patterns, and relationships within unlabeled datasets, and is frequently used for clustering, pattern discovery, and dimensionality reduction. In contrast, supervised learning relies on labeled data to learn explicit input–output mappings and is widely applied when a clearly defined prediction target exists. Deep learning methods extend these capabilities by learning hierarchical feature representations directly from data, enabling more expressive modeling of complex, high-dimensional inputs such as neuroimaging or biomedical signals.

### 3.1. Linear Discriminant Analysis

**Figure 2:**
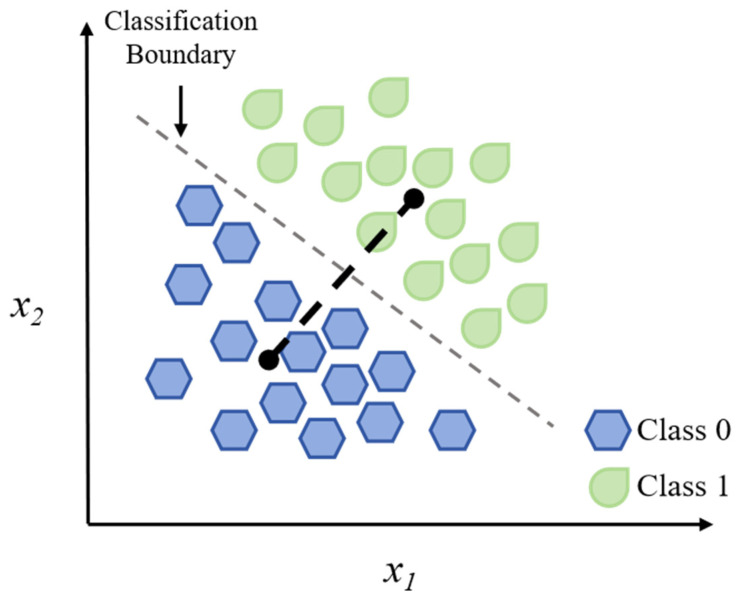
Schematic diagram of the LDA algorithm. The dush lines in the figure represent the classification boundaries.

Linear Discriminant Analysis (LDA) is a mature supervised technique for dimensionality reduction and classification, visually represented by its projection onto a discriminative axis (Figure 2). For food safety detection, the LDA method demonstrates good performance in multiple tasks, such as the online diagnosis of subclinical mastitis in milk, the classification of sea bass filet freshness using Raman spectra, and the identification of duck meat adulteration in beef [41,42,43]. Combined with other analytical techniques, the robustness of LDA is further strengthened. For instance, its integration into multimodal spectroscopy for predicting fish freshness and its application with hyperspectral imaging to identify milk adulteration with 100% validation accuracy confirm its competence in analyzing high-dimensional, multisource information [44,45]. The key advantages of LDA include its computational efficiency and the generation of interpretable, linear decision boundaries. However, the success of this approach is critically dependent on its strong underlying assumptions that data follow Gaussian distributions and share common covariance across classes, which can limit its applicability to complex, real-world food matrices where these assumptions are often violated.

Although there are some advantages from an explainability and computation point of view, the success of this approach is based on two strong assumptions about the data, namely, that the data come from Gaussian distributions and have common class-covariance. Even though the above-mentioned approaches have several drawbacks, they have been successfully applied in different food safety contexts, such as microbiological studies, detection of chemical hazards, and authentication tests, due to their properties of generating understandable decision boundaries and solving different kinds of problems efficiently.

### 3.2. Naive Bayes

**Figure 3:**
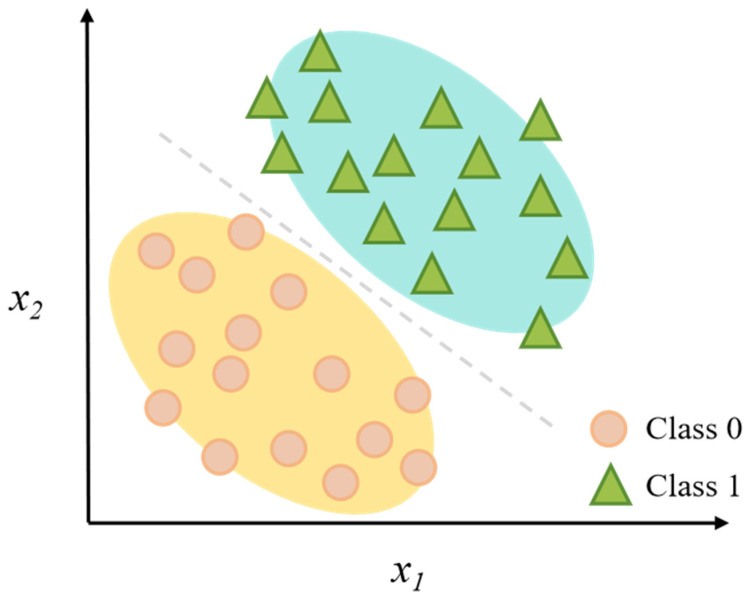
Schematic diagram of the Gaussian Naive Bayes classification algorithm.

**Figure 4:**
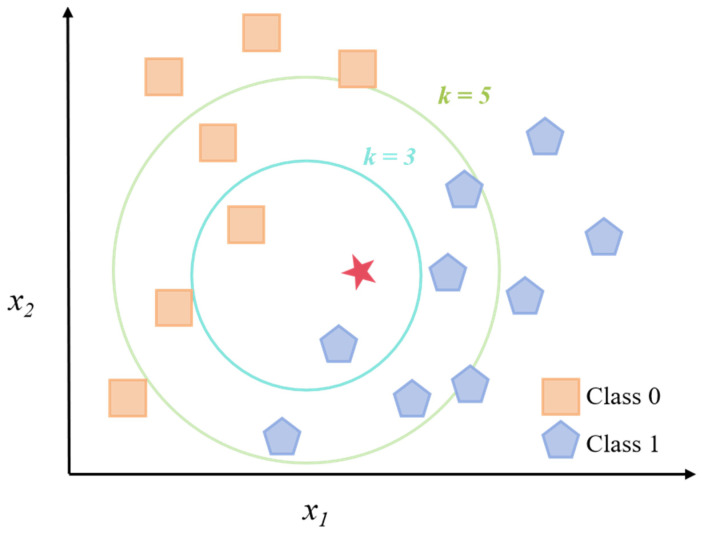
Schematic diagram of K-NN algorithm. The red star in the figure represents the critical center point.

The Naive Bayes (NB) algorithm is based on Bayes’ theorem and assumes that any two features are independent of conditional probability in any category term (Figure 3) [46]. Based on this assumption, the calculation is greatly simplified; even if there is little training data, NB can still perform good prediction performance and be calculated efficiently as well as implemented simply [47]. However, if there are highly correlated features, the assumption of independent attributes will degrade predictive accuracy greatly [48]. There have been many variants proposed from different perspectives so far. These variants are designed according to the different statistical characteristics of features, primarily including: Gaussian NB for continuous numerical data; Multinomial NB for discrete count data (e.g., word frequencies); Complement NB as an adaptation of Multinomial NB for imbalanced text datasets; Bernoulli NB for binary/Boolean features (e.g., word presence or absence); and Categorical NB for categorical discrete data [49]. Among them, the Gaussian Naive Bayes (GNB) algorithm (Figure 4) has demonstrated good performance for many tasks related to food safety risk assessment. For example, Vannya et al. [50], Soon et al. [51] adopted a kind of Bayesian method to forecast food fraud categories and spots of fake food ingredients, illustrating that NB-based approaches possess good interpretability and transferability for solving difficult food identification problems.

In a study on supply chain vulnerability, Bouzembrak et al. [52] applied a GNB model to assess the risk of food fraud in the spice supply chain. The researchers combined several data sources from the EU’s Rapid Alert System for Food and Feed (RASFF), such as historical records of infringements, and Product characteristics and geographical sources. This integration demonstrates GNB’s efficiency in analyzing complex, multidimensional food safety data. This dataset forms the basis for comparing GNB’s performance with more complex integration methods. Although GNB offers an explanatory and efficient method of calculation, its effectiveness is influenced by the properties of the character distribution and the hypothesis of the normality of continuous variables, especially when processing various supply chain data.

### 3.3. K-Nearest Neighbors

The K-Nearest Neighbors (K-NN) algorithm (Figure 4) is an instance-based, non-parametric learning method widely used in classification and regression tasks. It works so that data points with similar characteristics often belong to the same category. For classification, the labeling of the new sample is determined based on a predefined distance measurement (e.g., Euclidean distance or Manhattan distance), which is determined by the majority of K’ s nearest neighbors in the character space. The choice of K is crucial because it balances a compromise between model deviation and variance. Although KNN is easy to implement and does not require clear training phases, is challenging in terms of computational efficiency on large datasets and sensitive to dimensional disasters, which makes the selection of features and the reduction in dimensions an indispensable pre-processing step in many applications [53].

In food safety risk assessment, the K-NN algorithm was successfully used in spectral analysis and rapid contamination inspection [54]. For example, one study applied K-NN together with flexible Surface-Enhanced Raman Spectroscopy (SERS) substrates to identify pesticide residues in fruits and vegetables [55]. The researchers first used PCA to reduce the data dimensionality before performing the KNN classification. This method allows for high sensitivity and portability and allows for effective differentiation of contaminated and uncontaminated samples based on the spectral Characteristics. These studies demonstrate the advantages of K-NN in the analysis of high-dimensional spectral data and its potential for on-site food safety testing [56].

In addition, the K-NN algorithm has its unique features in predicting microbial hazard risks. In one research work, K-NN combined with a novel flexible SERS substrate was used for the detection of pesticide residues in fruit and vegetable samples [57]. It demonstrated good predictive performance for predicting the samples’ contamination risk grade after proper feature scaling, and the best neighbor number k was picked out. And its simple structure and easy interpretation features make it an ideal starting model for preliminary prediction and a reference standard for evaluation of complex algorithms [58].

### 3.4. Support Vector Machine

**Figure 5:**
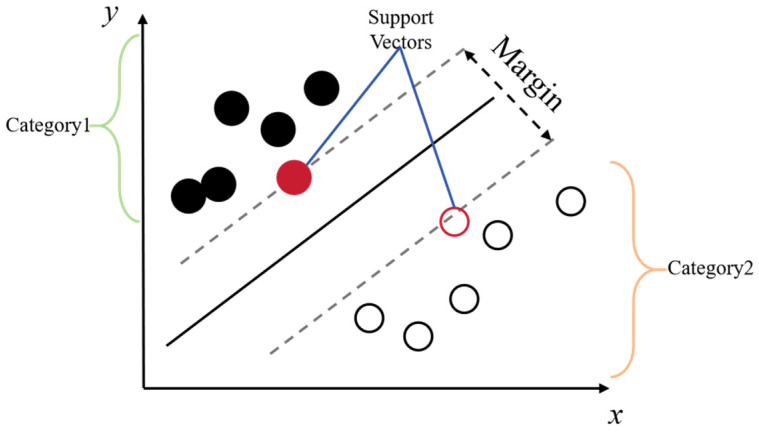
Schematic diagram of the SVM model. The black dots in the figure represent category 1, and the white dots represent category 2.

Support Vector Machines (SVM) represent a powerful supervised learning algorithm (Figure 5) that constructs an optimal hyperplane in high-dimensional feature space to maximize separation between classes [59,60]. For food safety risk assessment, SVM demonstrates exceptional versatility across multiple domains. It shows promise in constructing comprehensive evaluation models that analyze risk across the entire food supply chain from production to consumption [61]. More specifically, SVM excels in spectral data analysis, achieving strong performance in detecting biotoxins such as aflatoxin B in almonds using fluorescence spectroscopy [62], and in determining heavy metal concentrations like mercury speciation in squid [63].

The algorithm’s strengths include its solid theoretical foundation, robustness in high-dimensional spaces, and good generalization capability with limited samples. These characteristics make SVM particularly valuable for various food safety applications involving spectral signals, supply chain information, and chemical measurement data. However, SVM performance is critically dependent on appropriate kernel selection and parameter tuning, and its computational efficiency decreases significantly with large datasets. While deep learning approaches have surpassed SVM in some complex image-based tasks, SVM remains a reliable and interpretable choice for many classification problems in food safety, especially those involving spectral data and structured features.

### 3.5. Random Forest

**Figure 6:**
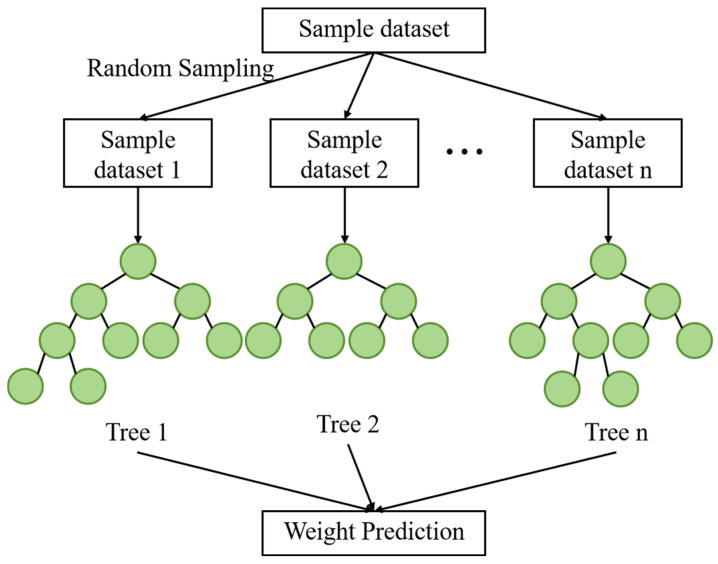
Schematic diagram of the Random Forest model.

Random Forest (RF), as a classical ensemble learning algorithm, operates by constructing multiple decision trees and aggregating their predictions for final results (Figure 6) [64]. In food safety risk analysis, RF’s prominent strength lies in its ability to handle high-dimensional data while providing intrinsic feature importance evaluation, which offers a convenient means to discover key risk factors from complex food matrices.

The method has demonstrated excellent performance in a comprehensive safety assessment. For grain resources, an AHP-RF model incorporating eight chemical contaminant indexes (heavy metals and mycotoxins) achieved a prognostic correlation coefficient exceeding 0.99, providing highly consistent risk projections [65]. Similarly, in supply chain risk prediction, a nationwide study of freshwater products based on over 300,000 samples demonstrated RF’s outstanding performance, with the model achieving 75.4% sensitivity and 78.0% accuracy while identifying five critical risk dimensions, including supply chain nodes, sampling areas, and environmental conditions [66].

RF’s key advantages include its robustness against overfitting, ability to handle complex nonlinear relationships, and provision of feature importance rankings. These characteristics explain its widespread adoption in processing complex food safety data [67]. However, the main limitations involve computational resource requirements with very large datasets and the inherent trade-off between improved accuracy and reduced interpretability compared to a single decision tree. Despite these limitations, as food supply chains grow more complex and data volumes increase, RF remains a fundamental algorithm for building intelligent early warning systems in food safety.

### 3.6. Gradient Boosting

**Figure 7:**
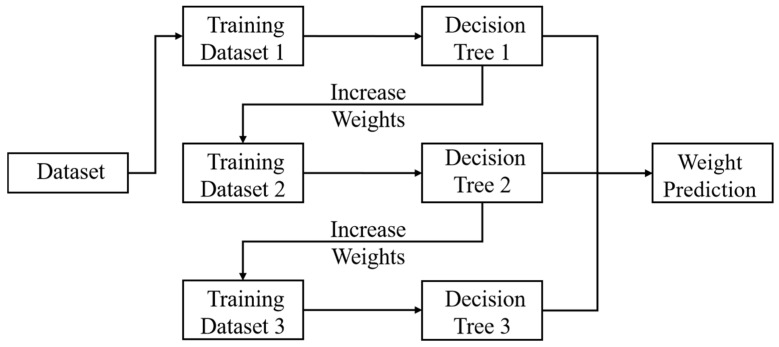
Schematic diagram of the Extreme Gradient Boosting model.

The Gradient Boosting is a powerful ensemble learning method that can build models step by step (Figure 7). Each new model focuses on correcting the errors of previous models [68]. This sequential method typically allows for higher accuracy than a single model or other integrated methods. It is particularly effective in processing complex and high-dimensional data common in food safety research.

Under the context of large-scale public health protection, the integrated method based on tree models with gradient boosting has good development prospects in the construction of early warning systems [69]. One study used human salmonellosis case numbers and food surveillance data as examples; the results showed that compared with general decision trees, the Gradient Boosting model had better performance. The model found key predictor variables, i.e., the positive rate of Salmonella in foods, and the positive rate of Salmonella in ready-to-eat milk and pork products.

The flexible and powerful prediction ability of gradient boosting makes it become the core method based on which modern food safety risk evaluation is made [70]. Researchers have successfully applied it to chemical contaminant quantification, microbial growth prediction, and supply chain risk management [71]. Its strong ability to model complex nonlinear relationships allows it to deliver reliable predictions in diverse analytical contexts. In the future, as the global food system continues to develop in a complex way, gradient boosting will still be an important means and method to build smart and databased food safety governance systems.

## 4. Deep Learning

Recently, DL has emerged as one of the most revolutionary and representative methods of artificial intelligence, providing efficient tools for analyzing complex data and making smart decisions. Different from traditional ML, DL uses multi-layered neural models, which are able to learn the hierarchical representations of input signals automatically. By taking advantage of nonlinear interactions and multiplex interactions among features or neurons, we can conduct the joint training process for feature engineering and model training, i.e., an end-to-end training framework. Hierarchical representation is beneficial for dealing with the problems of high dimensionality, structurelessness, and multiple modalities of real-world data.

In the aspect of food safety risk assessment, the emergence of the DL method provides a new solution idea for modeling the food safety system. Food safety data usually have high heterogeneity, time shift changeability, and complex space correlation, which brings difficulties for traditional algorithm modeling and mining potential associations. Different structures of models in DL, such as convolutional models, recurrent models, attention mechanism-based models, chart models, and enhanced learning models, can jointly model and extract characteristics from image spectra, time series signals, text, and so on, ranging from single-task auditing to full-aspect risk provision from a systemic perspective. With the continuous improvement of data acquisition speed and computing power, DL has become one of the most prominent research hotspots to boost the intelligence level of food safety supervision and control.

### 4.1. Convolutional Neural Networks

**Figure 8:**
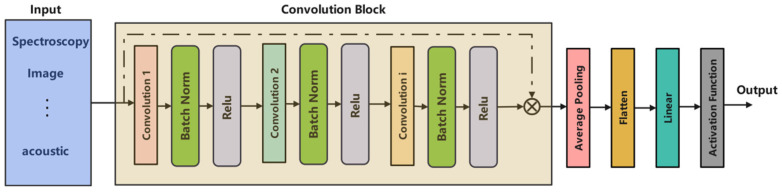
Schematic diagram of a typical Convolutional Neural Network (CNN) architecture.

Conventional Neural Networks (CNN) are one of the most basic and widely used architectures in DL and are known for their powerful capabilities for automatic character extraction and hierarchical representation of known patterns (Figure 8). The core concept of CNN is to use local sensing fields and parameter distribution, so that models can capture spatial or spectral correlations in high-dimensional data. As a rule, a CNN consists of a convolution layer, an activation layer, a pooling layer, and a complete connection layer. As shown in Figure 8, the conversion operation extracts local features; Nonlinear activation functions such as ReLU introduce nonlinearity. Sampling under the pool layer to reduce overmatching and calculation costs. The entire connection layer integrates features for the final classification or regression.

With the development of the DL architecture, CNN has experienced many structural innovations. From earlier architectures like LeNet and AlexNet to deeper variants like VGG, ResNet and DenseNet have continuously developed the function extraction capability and stability of gradient propagation through mechanisms such as Deep, Improved residual connectivity, and batch naturalization. Encoder–decoder architectures such as U-Net allow for space reconstruction with fine-grained size and representation in symmetrical convolution and sampling paths. Several scales that further extend CNN to semantic segmentation and intensive forecasting tasks.

Recently, the research community has further promoted the development of CNN models in multiscale, multispectrum, and interaction learning aspects. Park et al. [72] have come up with a deep convolutional segmentation framework based on the integration of spatial–spectral features for joint feature learning. Wang et al. [73] adopted one-dimensional convolutional layers to model sequential signals at different scales, showing that CNN operations can also be applied to frequency domain feature transformations. Hassan et al. [74] designed a hybrid deep convolutional structure with interaction blocks as an additional component to improve its adaptability and responsiveness. Chen et al. [75] proposed a Residual-based Convolutional Classifier for feature discrimination in the output space of neurons at deeper layers. Chakravartula et al. [76] implemented a multi-channel convolutional architecture for spectral data modeling, effectively capturing latent relationships across high-dimensional signals. Peng et al. [77] took advantage of ResNets with 1D CNN architecture and succeeded in predicting the copper stress grade of rapeseeds by hierarchically extracting representations from input spectra while preserving useful original information by shortcut connections in the deep layers. As for quantitative prediction, Guo et al. [78] reported a 2D CNN model for predicting AFB1 content in Aspergillus-infected peanuts using near-infrared hyperspectral imaging datasets, where the pixel values within each channel were considered as features for deep spatial–spectral feature learning [79].

CNN has hierarchical representation learning ability; it can automatically detect complex spatial/spectral patterns from raw data, reduce manual feature characterization, and combine the advantages such as good scalability, strong generalization ability, and fast convergence speed, so that CNN becomes an important and basic deep-learning framework. With the development of lightweight structures like MobileNet, EfficientNet series, and more emphasis on the interpretability study of models, we believe that CNN will still be the core supporting technology in the construction of various intelligent analysis and reasoning systems in future intelligent adaptability computation.

### 4.2. Recurrent Neural Networks

**Figure 9:**
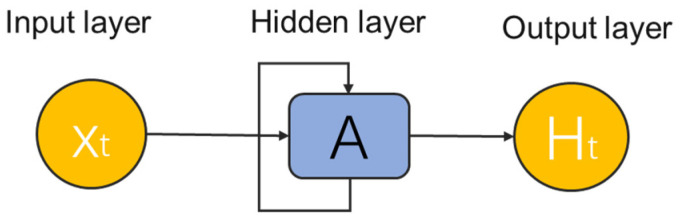
Schematic diagram of a typical RNN architecture.

Recursive neural networks (RNNs) are a class of DL architectures designed specifically for modeling sequence and time dependencies in data. Unlike traditional forward-feeding neural networks, RNNs combine a cyclic connection that allows information to exist over time (Figure 9). The hidden state of each time step captures context information from previous inputs, so that the network maintains a form of memory and dynamic, can effectively simulate behavior that changes over time. This structural property makes RNN particularly suitable for tasks that include sequence modeling, time signal processing, and context perception prediction.

However, it is known that standard RNNs have problems with disappearance and explosion gradients when they propagate backwards through long sequences, which limits their ability to replicate. capture long-term dependencies. To overcome this limitation, the LSTM network was introduced. It reinforces the conventional circuit architecture through door control mechanisms (input, forget, and output doors) that regulate the flow of information within the cell state. These doors allow the model to selectively retain or discard time information to effectively reduce gradients and enable remote dependency learning. A related variant, the door control loop unit (GRU), simplifies this design by merging entrance and forget doors into an update door and reduces parameter complexity, while maintaining comparable performance, which is particularly advantageous in a resource-constrained environment.

With further development and improvement of the recurrence mechanism, various improved recurrence models were proposed later. Wu et al. [80] propose an attention mechanism-enhanced recurrent framework, which adaptively weights important features along with long-time sequences and strengthens global dependence among samples. Nagamalla et al. [81] detect and classify milk adulteration by using RNN and upload it through Internet of Things (IoT). Deng et al. [82] showed that RNN can well capture the sequence dependency information contained in Raman spectrum signals, which is beneficial for qualitative and quantitative analysis of aflatoxin content in edible oil. Zhu et al. [83] further introduce RNN-based structures to optimize the reconstruction of spectra and realize high-precision aflatoxin detection with smaller-sized models. Wang et al. [84] proposes a BiLSTM-based fusion network for Hyperspectral Imaging (HSI) and optimizes this model to further promote the pixel-wise detection performance of aflatoxin in peanut kernels.

Furthermore, bidirectional recurrent networks (BiRNNs) extend the model’s temporal context by processing sequences in both forward and backward directions, while stacked or multi-layer RNNs deepen the temporal abstraction hierarchy to enhance representational capacity. With the advent of transformer architecture, hybrid models such as Conv-LSTM and Attention LSTM gained attention, combining the cyclical properties of RNNs with the parallelization of transformer-based designs and the global attention benefits

Overall, RNNs and their extensions (LSTM, GRU, and BiRNN) form the foundational framework for temporal and sequential modeling in DL. Their inherent ability to capture dynamic dependencies, learn time conversions, and code sequence states makes them indispensable for time-dependent forecasting tasks. Although Transformer-based models have dominated long-distance learning in recent times, recursive networks are still very effective at problems that affect continuity. require small sample adjustment or real-time reasoning. With the continuous advances in hybrid architectures and lightweight recursive models, RNN-based frameworks play a central role in deep-time modeling and online sequence design.

### 4.3. Transformer and Attention Mechanism

**Figure 10:**
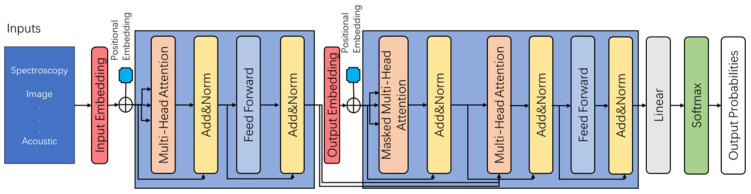
Schematic diagram of a typical Transformer architecture.

The Transformer architecture has become one of the most influential advances in DL and has fundamentally redefined the modeling of sequence and context dependencies (Figure 10). The core innovation consists of an attention mechanism that dynamically assigns importance to the various input characteristics, so that the model can selectively focus on most of the data. Unlike recurrent architectures that rely on step-by-step computation, the Transformer enables full parallelization through self-attention, achieving efficient global dependency modeling and significantly improving representational depth and computational scalability. Unlike a circuit architecture that is based on step-by-step calculations, Transformer enables complete parallelization through self-attention. It provides efficient global dependency modeling and improves representation depth and computational scalability.

A standard transformer consists of an encoder–decoder framework in which each component contains a multi-head self-attention layer and a position forwarding network. The multi-head attention mechanism projects the inputs into several sub-spaces and calculates the attention distribution in parallel, so that the network can capture information across multiple relationship scales. The Positional encoding can maintain the relationships in the data. By stacking multiple attention and forwarding layers, Transformer can model complex contextual relationships and provide advanced semantic representations in a large Abstract feature space.

To improve efficiency and versatility, various improvements and extensions to the transformer architecture have been proposed. Aghamohammadesmaeilketabforoosh et al. [85] developed a Vision Transformer (ViT)-based framework to convert visual data into continuous patch embeddings and enable global function modeling through self-attention. Shao et al. [86] implemented a Transformer-based model for intermediate-level fusion of VNIR and SWIR hyperspectral data, enabling robust classification of moldy soybeans through global spectral dependency modeling and self-attention-driven feature integration. Wu et al. [87] proposed a SpecTransformer model for enabling accurate quantification of pesticide residues in cherry tomatoes through deep spectral feature learning via multi-head self-attention and positional encoding in a 1D spectral sequence. Chen et al. [88] developed an MLP-Transformer hybrid model for the identification of apple moldy core by capturing long-range dependencies and cross-modal interactions through self-attention mechanisms. Wang et al. [89] proposed a 1D-MCFViT model. They combined Vision Transformer with multi-scale convolutional fusion. Guo et al. [90] further demonstrated that integrating convolutional fusion and Transformer modules enhances model generalization on complex spectral data. Kim et al. [91] proposed a Compact Convolutional Transformer (CCT) for classifying wheat contaminated by multiple mycotoxins by integrating convolutional feature extraction with Transformer attention. These models integrate the local sensitivity of convolutional filters with the global dependency modeling of attention and achieved efficient fusion of localized and contextual information. For tasks with a long sequence, lightweight variants such as Performer, Longformer and Linformer use a strategy of low layout or thin attention, to reduce computational complexity while maintaining modeling capability.

In summary, the introduction of Transformer and attention mechanisms represents a paradigm shift from local feature extraction to global relational modeling in DL. Their ability to capture long-range dependencies and context-aware interactions has made them indispensable for a wide range of sequence, vision, and multimodal learning tasks. With ongoing innovations in architectural design and computational optimization, Transformer-based models are increasingly becoming the cornerstone of modern neural network architectures. Looking ahead, the integration of self-supervised learning and cross-modal attention mechanisms is expected to further enhance their adaptability and interpretability, paving the way for broader applications in complex system modeling and intelligent decision-making.

## 5. Application of Machine Learning and Deep Learning in Food Safety Risk Assessment

In this section, we will introduce the application of ML and DL technology in the key links of food safety risk assessment in four main categories of food safety risks: biotoxins, heavy metal pollution, Pesticide and veterinary drug residues and microbial hazards (Table 1, Table 2, Table 3 and Table 4). Food safety risks often come from multiple sources and show dynamic and complex characteristics. The existing methods mainly depend on statistical analysis and expert experience, which make it difficult to deal with high-heterogeneity data, and unable to handle nonlinear relationships and timely warnings [92]. As mentioned in previous chapters, a variety of calculation methods have already evolved to address these challenges. These include basic algorithms such as simple bayes and supported vector machines, integrated learning technologies such as random forests and gradient augmentation, as well as advanced DL architectures that can automatically learn the layer-based representation of features. These include convolutive neural networks, recursive neural networks and transformers [93].

### 5.1. Mycotoxins Risks

Mycotoxins are ubiquitous contaminants in the food supply chain, inducing severe health effects ranging from allergies to carcinogenesis even at trace levels [94]. Among the various types, Aflatoxin B1 (AFB1) and Deoxynivalenol (DON) pose the most significant public health threats [95]. AFB1 is a potent carcinogen prevalent in legumes and nuts, while DON is a thermally stable contaminant in cereals that persists throughout processing [83,96]. To mitigate these risks, global agencies enforce strict limits. The JECFA and FDA set a 1 mg/kg (ppm) guidance level for DON in finished foods, while Health Canada mandates stricter limits of 0.6 ppm for infant foods [97,98]. Similarly, the EU enforces Maximum Residue Limits (MRLs) for AFB1 in dairy feed at ≤5 μg/kg [99]. Given the heterogeneous distribution of these toxins and the need for parts-per-billion (ppb) level sensitivity, traditional methods often lack the necessary efficiency. Consequently, machine learning-based approaches have become essential for achieving the rapid, precise detection required for effective early warning systems.

Kim et al. [91] utilized a Compact Convolutional Transformer (CCT) model to detect early mycotoxin contamination in stored wheat caused by deoxynivalenol (DON) and aflatoxins. This approach facilitates the classification of wheat into healthy, incipient, and contaminated classes based on CO_2_ respiration rates and visual mold formation, achieving an overall accuracy of 83.33%. Furthermore, the method effectively identified the contaminated class with high performance metrics (precision of 1.0 and F1-score of 0.95), while the distinction between healthy and incipient stages (F1-scores of 0.81 and 0.75, respectively) requires further improvement.

Guo et al. [100] coupled a multi-scale attention transformer (MSAT) with HSI to classify Aspergillus flavus contamination in peanut kernels. By employing a sophisticated attention mechanism that captures features at varying resolutions, the MSAT model achieved a test accuracy of 98.42% in distinguishing healthy kernels from those contaminated with aflatoxigenic fungi.

Siripatrawan and Makino [40] utilized machine learning-assisted HSI to classify Penicillium contamination levels in brown rice. Among the algorithms tested, the Gaussian Support Vector Machine (SVM) yielded the best performance with an accuracy of 93.4%, successfully identifying contamination levels as low as 5% that were invisible to the naked eye. This model surpassed other classifiers, including linear DFA (67.6%), linear SVM (76.0%), and quadratic SVM (81.4%).

From a performance perspective, CNN models [78,84] show strong capabilities in automatic feature extraction, while the emerging Transformer architecture [87,90,91], by virtue of its self-attention mechanism, outperforms in capturing global spectral context and achieving pixel-level precise localization, improving classification or localization accuracy by 3–5% in certain tasks. A notable trend is the multi-modal data fusion of macro and micro features (e.g., fused VNIR, HMI, and SEM [90]), and hybrid models combining CNN and BiLSTM [84], which aim to more comprehensively characterize the complex features of toxins by synergizing spatial and spectral, spatial and temporal information. However, a key challenge lies in the heavy reliance of these high-performance models on high-quality annotated data and their high computational cost, hindering their real-time deployment on production lines. Future research must focus on developing lighter-weight models and exploring few-shot learning paradigms to address the practical problem of data scarcity.

**Table 1:** Applications of Machine and Deep Learning in the Detection of Mycotoxins Toxins.

Product	Purpose of Study	Data	Algorithm/Model	Output	References
Herbs and Spices	To prioritize products and hazards for monitoring across the supply chain	RASFF and Dutch national monitoring data (2005–2014)	NB	ACC = 80%	Bouzembrak, Y. et al. (2016) [101]
Almonds	Non-destructive detection of aflatoxin B contamination	Fluorescence spectra of almond samples with known aflatoxin levels (2.7–320.2 ng/g)	SVM	ACC = 94%	Bertani, F. R. et al. (2020) [62]
Wheat	Predict early contamination of deoxynivalenol (DON) and aflatoxins	RGB images and CO_2_ respiration rate data	CNN, Transformer	ACC = 83.33%	Kim, et al. (2024) [91]
Rice grain	Classification of fungal contamination in brown rice	HSI data	PCA, SVM	ACC = 93.4%	Siripatrawan, et al. (2024) [40]
Peanuts	Propose a novel aflatoxin B1 (AFB1) detection method.	HSI data	Autoencoder, LSTM, PCA	ACC = 98.3%	Zhu et al. (2024) [83]
Peanut	Detect fungal contamination caused by Aspergillus flavus	HSI data	Transformer	ACC = 98.42%	Guo, et al. (2024) [100]
Soybean	Detect fungal contamination caused by Aspergillus flavus	VNIR (400–1000 nm) and SWIR (1000–2500 nm) HSI data	CNN, Transformer, SVM, PCA	ACC = 97.52%	Shao, et al. (2025) [86]
Maize and Peanuts	Early detection and quantitative prediction of aflatoxin B1 contamination	Bioluminescence signals from whole-cell biosensors; AFB1 levels measured by HPLC	XGBoost	R^2^ > 0.9	Sun, L. et al. (2025) [102]
Food (general)	Screening fungal toxin characteristics to predict toxicity	Molecular descriptor representation and toxicity value of mycotoxins	HCA, K-means, SVM, LDA, Neural Networks	-	Cova, et al. (2025) [103]
Maize silage	Detect aflatoxin B1 (AFB1) content	HSI data	CNN	R^2^ =0.9458	Guo, et al. (2025) [78]
Peanut	Detect Aspergillus flavus contamination	VNIR hyperspectral imaging (400–1000 nm), Hyperspectral Microscopic Imaging (HMI), Scanning Electron Microscopy (SEM) images	CNN, Transformer	ACC = 100%	Guo, et al. (2025) [90]
Peanut	Pixel-level detection of aflatoxin B1 (AFB1)	HSI data; spectral curve data	CNN, LSTM	ACC = 94.92%	Wang, et al. (2025) [84]
Peanut	Detect aflatoxin B1 (AFB1) contamination	Visible near-infrared (VNIR) hyperspectral imaging data (400–1000 nm)	CNN, Transformer	ACC = 92.6%	Wang, et al. (2025) [89]
Edible oil	Rapid, non-destructive detection of aflatoxin B1 (AFB1) contamination level	Raman spectroscopy data	CNN, RNN	ACC = 100%	Deng, et al. (2025) [82]

### 5.2. Heavy Metal Pollution

Detection and evaluation of Heavy Metal Contamination in Food is an important issue in current food safety governance. Lead (Pb), Cadmium (Cd), Mercury (Hg), Arsenic (As) are all toxic heavy metals with accumulation effects and persistence in the food chain, which seriously affect human health. The traditional detection method, like Atomic Absorption Spectroscopy and Inductively Coupled Plasma Mass Spectrometry, has higher analysis precision but will take a long time, be costly, and not be applicable for such large-scale/real-time detection demands. Therefore, ML/DL-based methods are proposed as the promising alternative ways that can realize fast, non-destructive, and smart detection in multiple kinds of food and environmental sample detection scenarios.

With the development of DL methods in recent years, two advantages are mainly reflected, which are the efficiency and accuracy of prediction for heavy metals. CNN can extract hierarchical spatial spectral feature representations. To be specific, it can obtain the high-level abstraction of input samples by learning representation layers layer-by-layer, in terms of different concentration levels of target elements. Therefore, the model can identify and classify the soil pollution grade through such characteristics. For instance, Peng et al. [77] predicted copper and lead stress in oilseed rape leaves based on hyperspectral image data using CNN; meanwhile, good prediction performance was still obtained when faced with changes in the growth environment. In a separate study, the results showed that the risk assessment order was Cd > Ni > Cu > Pb > Cr > Zn [104]. In addition, the WT–SAE network is an improvement based on traditional methods. Compared with previous models, WT–SAE not only performs better in the aspects of denoising and extracting nonlinear features of spectra but also estimates the content of Pb in plants more accurately.

Hybrid modeling strategies that combine DL with traditional regression methods have also shown strong performance in quantitative analysis [105]. For instance, the Transfer Stacked Contractive Autoencoder combined with Support Vector Regression (T-SCAE + SVR) has good predictive ability and can be generalized well when predicting heavy metal contents based on images collected from experiments under different growth conditions. The hybrid framework combines two advantages: deep models provide high-level abstract feature extraction, while classical regression acts as an explainer of the relationships between target and features.

VM-based methods are still an important means of spectral and chemometric analysis, especially when the amount of data is not large; when they are used with fluorescence spectra, near-infrared spectra, etc., they can well be applied to determine pollution sources, origin identification, and so on. For example, one paper has successfully determined the level of mercury contamination in aquatic products by using this method [106]. It shows again that, regardless of the type of data as well as other analysis conditions, the model built based on SVM still performs well in terms of its stability and anti-jamming ability.

In general, the combination of ML, hyperspectral imaging, and chemometrics has upgraded heavy metals detection from traditional methods into an intelligent, fast-speed, large-scale analysis method [107]. Future research will likely emphasize multimodal data fusion that combines spectral, spatial, and physicochemical features. It will also focus on integrating interpretable AI models into regulatory monitoring systems to enable continuous and automated surveillance of heavy metal risks throughout the food supply chain.

In heavy metal pollution detection, the technological trajectory shows an evolution from traditional spectral analysis towards deep feature learning. Comparative analysis reveals that although traditional methods like SVM [63] remain effective in specific scenarios, deep learning models represented by CNN and Autoencoders [77,108,109,110] demonstrate clear advantages in extracting deep, nonlinear features related to heavy metal stress from fluorescence hyperspectral images, achieving higher prediction accuracy. An innovative direction is the combination of the feature extraction capability of deep learning with the interpretability of classical regression models (like SVR) [108,111]; this hybrid strategy enhances model understanding while maintaining performance. However, a limitation of current research is that most studies are conducted in controlled environments, and the practical robustness of the models faces severe challenges when dealing with complex and variable field environments, different crop varieties, and soil matrices. Future work should prioritize the model’s cross-environment generalization capability and integration with field sensors.

**Table 2:** Applications of Machine and Deep Learning in the Heavy metal pollution.

Product	Purpose of Study	Data	Algorithm/Model	Output	References
Lettuce	Extracting compound heavy metals detection deep features of lettuce leaves	Visible near-infrared (400.68–1001.61 nm) hyperspectral image	Autoencoder, SVR	R^2^ = 0.9319	Zhou, et al. (2020) [111]
Boletus mushroom	Assess whether cadmium (Cd) content exceeds safety limits	Fourier Transform Near-Infrared (FT-NIR) spectroscopy data	ResNet	ACC = 100%	Wang, et al. (2021) [112]
Oilseed rape	Prediction of lead (Pb) content	FHSI data (390 nm UV excitation)	Autoencoder, SVR	R^2^ = 0.9388	Zhou, et al. (2023) [109]
Oilseed rape	Prediction of lead (Pb) content under silicon-present and silicon-absent conditions	FHSI data (390 nm UV excitation)	Autoencoder, SVR	R^2^ = 0.9467	Zhou, et al. (2022) [108]
edible oils	Predicting heavy metals in edible oils	microwave data	ResNet	R^2^ = 0.9605	Deng, et al. (2024) [113]
Oilseed rape	Classification of copper (Cu) stress levels	HSI data	CNN	ACC = 98.15%	Peng, et al. (2025) [77]
Squid	To develop a method for mercury determination and geographical origin traceability.	THg and MeHg concentrations in 50 squid samples from Mediterranean and Atlantic.	SVM	ACC = 100%	Piroutková, M. et al. (2025) [63]

### 5.3. Pesticide and Veterinary Drug Residues

Analysis of pesticide residue and veterinary drug residue is another aspect involving testing of pesticides and residues of veterinary drugs that belongs to food safety risk identification. Pesticide residue/veterinary drug residue exists widely in agriculture and may cause great harm to humans. Traditional chromatographic and mass spectrometric methods have high analytical sensitivity but generally require complicated sample treatment and costly equipment and cannot be used for rapid or on-site detection; while ML/DL approaches provide another efficient alternative solution and realize rapid, non-destructive, and data-driven measurement based on spectra, images, and fluorescence signals.

DL models like 1D and 2D CNN are developing well in residue detection, which does not require manually pretreated data and has a good ability for learning representative spectra features from the original signal. In fruit and vegetable samples, CNN architectures have been used for detect imidacloprid and acetamiprid residues through visible and near-infrared (VIS/NIR) spectroscopy. Moreover, one-dimensional residual networks (1D-ResNet) and spectral convolutional models have shown better performance than traditional chemometric techniques such as PLS-DA and SVM in classifying contaminated samples [55]. These models effectively capture subtle spectral changes caused by pesticide molecules, resulting in improved detection accuracy.

Transformer-based architectures have further expanded analytical capabilities in this field. Through their self-attention mechanisms, Transformer models such as SpecTransformer can capture long-range relationships and complex feature interactions within spectral data. This design enhances both interpretability and robustness. The combination of convolutional layers and attention modules allows these models to extract local and global features simultaneously, improving generalization across different types of food samples. In addition to spectrum analysis, image-based DL is also widely used for the detection of antibiotic and pesticide residues by fluorescence and Raman imaging [114].

For pesticide and veterinary drug residue detection, algorithm development is closely linked to the trend of portable detection technology. Performance comparison shows that architectures like 1D-CNN and 1D-ResNet [115] are particularly suitable for processing spectral sequence data from portable spectrometers, and their end-to-end learning capability surpasses that of SVM and KNN [116], which rely on manual feature selection. Particularly noteworthy is the SpecTransformer model [87] designed for sequence data, which shows potential for capturing long-range spectral dependencies and achieves higher accuracy in quantitative analysis. A prominent trend is the focus on on-site rapid detection, such as combining handheld Raman spectrometers with CNN models for detecting formaldehyde and antibiotics [117,118]. However, a major gap in this field is that most existing studies target single or a few residues; the model’s recognition capability, anti-interference ability, and accurate quantitative analysis for the complex scenario of multiple pesticide co-residues commonly found in practical agricultural applications still require in-depth verification. The future development is mainly concentrated in three aspects: how to build a lighter model structure, how to realize multi-modal data fusion, and how to achieve XAI. This would further promote the generalization ability and robustness of models, realizing accurate, controllable, automatic, and traceable detection of pesticide/residue content from production to the circulation of food.

**Table 3:** Applications of Machine and Deep Learning in the Pesticide and Veterinary Drug Residues.

Product	Purpose of Study	Data	Algorithm/Model	Output	References
Chili pepper	Detection of imidacloprid and acetamiprid pesticide residues	VIS/NIR spectroscopy data (400–2498 nm)	CNN, SVM, KNN,	RMSE = 0.55	Ong, et al. (2023) [116]
Food	Rapid and user-friendly detection of tetracycline antibiotics (*TCs*)	Fluorescence images under 365 nm UV light from PVA aerogel sensor	ResNet	ACC = 99%	Chen, et al. (2024) [118]
Apple	To detect ten distinct types of pesticides	The fingerprints of ten pesticides	CNN	ACC = 100%	Wang, et al. (2024) [119]
Kumquat (Citrus japonica)	Detection of surface pesticide residues	VNIR spectral data	1D-ResNet, 1D-CNN, SPA-SVM	ACC = 97%	Dai, et al. (2025) [115]
Cherry tomato	Detection of thiophanate-methyl pesticide content	22-band spectral data from handheld spectrometer (210–1600 nm)	Transformer	R^2^ = 0.91	Wu, et al. (2025) [87]
Pacific white shrimp	Rapid and non-destructive detection of formaldehyde (FA) adulteration	Raman spectroscopy data	CNN	ACC = 84.40%	Wei, et al. (2025) [117]
grape	Detection of pesticide residues	images of grape samples	ResNet, EfficientNet	ACC = 83.17%	Saatçi, et al. (2025) [120]
bok choi	Detection and monitoring of pesticide residues in crops	The NIR spectral of bok choi with and without pesticide residue (chlorpyrifos)	CNN	ACC = 100%	Lapcharoensuk, et al. (2025) [121]

### 5.4. Microbial Risks

Microbial contamination is one of the most common and difficult problems in food safety globally. Microbial contamination may cause food spoilage, foodborne illness outbreaks, and huge economic burdens. Quick and precise detection of microorganisms can guarantee the quality of products and protect the public from the threat of pathogens.

The traditional microbiological method based on culture and/or biochemical tests is time-consuming, laborious, and not applicable for batch testing; while the development of ML/DL technology provides a new means for pathogen detection based on image/spectrum/sensor signal of microorganism [122].

CNNs have become the core of detecting microbial risks because they can learn complex visual and spectral features. In high-spectrum and fluorescence applications, CNN models can effectively distinguish between contaminated and clean samples. Advanced architectures such as EfficientNet and ResNeXt enable high accuracy in the classification of microbial corruption in meat and poultry products [123]. A semantic segmentation model, such as U-Net, further improves spatial positioning and enables precise pixel-level detection of bacterial colonies or contamination areas on the surface. food surface.

The Transformer-based models are increasingly used to integrate multimodal data sources, including visual, auditory, and spectral information [124]. They have shown strong performance in detecting early-stage fungal infections in fruits through hyperspectral fluorescence imaging. Combined CNN-LSTM models also offer advantages by modeling temporal changes in microbial growth, providing valuable insights into contamination dynamics over time.

The integration of IoT sensor networks with cloud-based AI frameworks has further enhanced microbial risk management [125]. These intelligent systems enable continuous data collection, real-time analysis, and automated decision-making. They represent an important step toward fully digital and self-adaptive microbial monitoring across the entire food supply chain.

In the field of microbial risk, DL is driving the transition of detection technology from culture-dependent methods towards rapid, non-destructive intelligent sensing [126]. Comprehensive analysis indicates that CNN models are the core tool for processing hyperspectral microscopic images [72,75], fluorescence images [127], and Raman spectra [73,128], achieving high accuracy in the identification and classification of various foodborne pathogens. Transformer models are beginning to be used for fusing multimodal data (e.g., acoustic signals and spectra) to capture richer contextual information. An important frontier involves combining object detection models (like YOLO) and semantic segmentation models to achieve automatic localization and counting of microorganisms, going beyond mere classification [129]. Despite this, a fundamental challenge remains: model performance is highly dependent on the scale and quality of training data. In real food systems, interference from background microbiota, the inherent complexity of the food matrix itself, and the requirement for detecting low concentrations of pathogens pose significant challenges to existing models. Developing models that remain robust against complex backgrounds is a key direction for the future.

**Table 4:** Applications of Machine Learning and Deep Learning in Microbial Risks.

Product	Purpose of Study	Data	Algorithm/Model	Output	References
Meat carcasses	Automatically identify and segment fecal contamination areas on meat surfaces	Fluorescence imaging (CSI-D device) video/image data	CNN	AUC = 99.54%	Gorji et al. (2022) [130]
Chicken	Non-destructive assessment of microbial spoilage	AC and DC images from SIRI	CNN, SVM	ACC = 76%	Olaniyi, et al. (2024) [131]
Strawberry	Early detection of gray mold (*Botrytis cinerea*) infection	FHSI data	CNN, ResNet	ACC = 96.86%	Chun, et al. (2024) [127]
Chicken rinse solution	Automated segmentation and identification of foodborne bacteria (*E. coli*, *Salmonella*, etc.)	HMI data	ResNet	ACC = 97.4%	Park, et al. (2023) [72]
Strawberry	Detect diseases and quality (gray mold, powdery mildew, ripeness)	RGB images	Transformer, ResNet	ACC = 98.4%	Aghamohammadesmaeilketabforoosh, et al. (2024) [85]
Food	Microscopic identification of 6 types of foodborne pathogens (*E. coli*, *S. aureus*, etc.)	Optical microscope images	CNN	6 kinds of foodborne pathogens with ACC ≥ 90%	Chen, et al. (2024) [75]
Apple	Detect and identify fungal spores	SERS data	CNN	ACC = 99.44%	Wang, et al. (2024) [73]
Food	Multiplex detection of foodborne pathogens	SERS data	CNN, Grad-CAM	ACC = 100%	Kang, et al. (2024) [128]
Apple	Online detection of moldy core disease (caused by fungi)	Acoustic signals and Vis-NIRS data	Transformer	ACC = 98.62%	Chen, et al. (2025) [88]
Fresh pork	Detect and visualize Escherichia coli contamination	HSI data	CNN, SVM	ACC = 87.50%	Liu, et al. (2025) [132]

## 6. Summary of Findings

The application of ML and DL technologies in food safety risk assessment significantly enhances detection accuracy, efficiency, and system intelligence. Classical models such as SVM, RF, and gradient boosting perform well in risk grading and contamination source tracking, especially when processing structured or spectral data. Their balance between predictive performance and interpretability makes them suitable for early warning systems and regulatory decision support.

DL further advances food safety analytics. CNNs excel in handling hyperspectral and imaging data for locating aflatoxins and other biotoxins. RNNs, including LSTM variants, provide effective solutions for time-series tasks in microbial growth and supply chain monitoring. Recently, transformer-based models have demonstrated strong capabilities in multimodal data integration, global context representation, and modeling complex hazard interactions.

Unsupervised learning methods such as K-means, hierarchical clustering, and PCA remain essential when labeled samples are limited, supporting anomaly detection, sample grouping, and dimensionality reduction. These techniques help extract intrinsic patterns and enable the construction of more reliable supervised risk prediction models.

Across major risk categories, ML/DL approaches have shifted food safety analysis from single-hazard detection to integrated intelligent prediction. CNN–Transformer hybrids support pixel-level localization and quantification of mycotoxins in hyperspectral images. FHSI combined with autoencoders and SVR enables noninvasive estimation of heavy metals like Pb and Cu. Portable spectrometers paired with 1D-CNN and SpecTransformer models facilitate rapid detection of pesticide and veterinary drug residues, while DL-driven hyperspectral and acoustic sensors support real-time microbial contamination prediction.

Despite these advancements, several challenges remain, including limited model interpretability, poor adaptability under small-sample conditions, difficulties in integrating heterogeneous multimodal data, and insufficient high-quality labeled datasets. This review summarizes progress in ML/DL-based food safety risk assessment, tracing developments from classical ML to modern DL and transformer architectures. We highlight algorithmic contributions to major hazard domains and outline remaining bottlenecks, emphasizing future directions in interpretability, dataset standardization, and multimodal fusion to guide the development of next-generation intelligent food safety supervision systems.

## 7. Classification, Limitations, and Future Directions of Machine Learning and Deep Learning Applications in Food Safety

ML and DL have significantly advanced food safety applications, supporting early risk detection through Bayesian models, autoencoders, and ensemble algorithms such as random forests and XGBoost. These methods enhance real-time monitoring and regulatory decision making, yet face challenges including high computational demands and difficulties in integrating heterogeneous data from diverse sources. In microbial and chemical hazard detection, ML/DL enables rapid, non-destructive identification of pathogens and contaminants using imaging, spectroscopy, optical sensors, and hyperspectral techniques. Although detection accuracy has improved, background microbial interference, environmental fluctuations, high equipment costs, and regulatory limitations still restrict large-scale deployment. ML also contributes to food fraud and adulteration detection through ensemble learning and spectral analysis, and neural networks can support freshness evaluation and spoilage prediction.

Despite these advances, ML applications are constrained by limited, inconsistent, and region-specific datasets, which undermine model generalization across different food matrices and processing environments. Moreover, the black-box nature of advanced neural models reduces interpretability and weakens user trust, especially among food safety professionals. Differences in data quality related to microbial and chemical hazards may introduce systematic biases, further affecting the stability and accuracy of model predictions.

Future research should prioritize developing standardized, publicly accessible datasets that encompass diverse hazard types and food products to improve model robustness and applicability. Enhancing interpretability features is essential as ML models become more complex, while deeper integration with spectroscopy, real-time monitoring platforms, and IoT systems will support automated, on-site food safety assessment. The combination of portable AI-enabled sensing devices, multimodal data fusion, and integration into HACCP frameworks will be crucial for achieving intelligent, interpretable, and real-time food safety management.

## 8. Conclusions

AI has become a pivotal force in modernizing food safety risk assessment. The findings of this review emphasize the revolutionary potential of ML and DL to transition the field from traditional post-detection methods to proactive, multi-source early warning systems. While classical algorithms such as SVM and RF remain effective for structured risk classification, this review highlights the superior capability of advanced DL architectures. CNNs for spectral feature extraction and Transformers for global context modeling achieve precise, non-destructive quantification of biotoxins, heavy metals, pesticide residues, and microbial pathogens. However, as these algorithms continue to augment detection sensitivity, bridging the gap between academic research and practical application becomes increasingly necessary. The integration of DL with non-destructive spectroscopic techniques (e.g., Hyperspectral Imaging, Raman) and IoT-based sensors offers a rapid alternative to conventional laboratory analysis, yet the “black box” nature of these models hinders their broad adoption in regulatory frameworks. To further enhance food safety governance, future research should prioritize the development of Explainable AI (XAI) and lightweight models deployable on portable devices, ultimately establishing a resilient, intelligent, and data-driven global food safety monitoring network.

## Data Availability

No new data were created or analyzed in this study. Data sharing is not applicable to this article.

## Abbreviations

The following abbreviations are used in this manuscript:

ACC	Accuracy
AI	Artificial intelligence
ADFPs	Animal-Derived Foods
AFB1	Aflatoxin B1
AHP	Analytic Hierarchy Process
BiLSTM	Bidirectional Long Short-Term Memory
BiRNN	Bidirectional Recurrent Neural Network
CCD	Charge-Coupled Device
CCT	Compact Convolutional Transformer
CNN	Convolutional Neural Network
DON	Deoxynivalenol
DL	Deep Learning
DT	Decision Tree
ELM	Extreme Learning Machine
EU	European Union
FA	Formaldehyde
FDA	Food and Drug Administration
FHSI	Fluorescence Hyperspectral Imaging
GNB	Gaussian Naive Bayes
GNN	Graph Neural Network
GRU	Gated Recurrent Unit
HACCP	Hazard Analysis and Critical Control Points
HCA	Hierarchical Cluster Analysis
HMI	Hyperspectral Microscopic Imaging
HSI	Hyperspectral Imaging
IoT	Internet of Things
JECFA	Joint FAO/WHO Expert Committee on Food Additives
K-NN	K-Nearest Neighbors
LDA	Linear Discriminant Analysis
LOF	Local Outlier Factor
LSTM	Long Short-Term Memory
ML	Machine Learning
MRLs	Maximum Residue Limits
MSAT	Multi-Scale Attention Transformer
NB	Naive Bayes
PCA	Principal Component Analysis
PLS-DA	Partial Least Squares Discriminant Analysis
RASFF	Rapid Alert System for Food and Feed
ReLU	Rectified Linear Unit
ResNet	Residual Network
RF	Random Forest
RNN	Recurrent Neural Network
SEM	Scanning Electron Microscopy
SERS	Surface-Enhanced Raman Spectroscopy
SIRI	Structured Illumination Reflectance Imaging
SVM	Support Vector Machine
SVR	Support Vector Regression
SWIR	Short-Wave Infrared
TC	Tetracycline
VIS/NIR	visible and near-infrared
VNIR	Visible Near-Infrared
ViT	Vision Transformer
WT–SAE	Wavelet Transform–Stacked Autoencoder
XAI	Explainable Artificial Intelligence
XGBoost	Extreme Gradient Boosting
